# Emotional problems and urinary incontinence in children from a UK cohort

**DOI:** 10.1016/j.jad.2025.04.020

**Published:** 2025-04-03

**Authors:** Carol Joinson, Mariusz T. Grzeda, Jon Heron

**Affiliations:** a Population Health Sciences, Bristol Medical School, University of Bristol, Oakfield House, Oakfield Grove, Bristol BS8 2BN, United Kingdom; b Galen Research, B1 Chorlton Mill, 3 Cambridge Street, Manchester M1 5BY, United Kingdom

**Keywords:** Emotional problems, Urinary incontinence, Enuresis, Daytime wetting, Children, Cohort study, ALSPAC

## Abstract

**Background:**

Emotional problems are more common in children with urinary incontinence (UI). This study examines (i) if UI is related to changes in emotional problems over time and (ii) if changes in emotional problems over time are related to the subsequent risk of UI.

**Methods:**

The study is based on data from 8188 children aged 6¾–9 years (50.7 % females) from the Avon Longitudinal Study of Parents and Children. Parents reported on their child’s UI (bedwetting and daytime wetting) at 7½ and 9½ years and emotional problems (Strengths and Difficulties Questionnaire) at 6¾ and 9½ years. We used a latent difference score model to examine (i) if presence of UI at 7½ years is related to the magnitude of change in emotional problems from 6¾–9½ years and (ii) if a change in emotional problems from 6¾–9½ years is related to the probability of UI at 9½ years.

**Results:**

UI at age 7½ was associated with a change (increase) in emotional problems over time (e.g. daytime wetting: unadjusted β = 0.205 (SE = 0.085), *p* < 0.001). The change was greater in children with both bed-wetting and daytime wetting (unadjusted β = 0.535 (SE = 0.103), *p* < 0.001). A change in emotional problems from 6¾–9½ years was related to the probability of UI at age 9½ (probit coefficient estimate = 0.145 (SE = 0.03), *p* < 0.001). Findings were robust to the inclusion of confounders.

**Conclusions:**

Children with UI experience increased emotional problems over time and changes in emotional problems were associated with subsequent UI. Emotional problems should be evaluated in children treated for incontinence.

## 1 Introduction

There is a robust association between psychological problems and childhood urinary incontinence (UI: daytime wetting and/or bedwetting (enuresis)). Evidence comes mainly from cross sectional studies making it difficult to determine if psychological problems are causes or consequences of UI (von Gontard et al., 2011). It is commonly believed that psychological problems in children with UI are due to the adverse impacts of UI on quality of life and self-esteem (Thibodeau et al., 2013). The perceived stigma of incontinence can cause children to feel abnormal and socially isolated, resulting in emotional problems including depression and anxiety symptoms (Whale et al., 2018). There is evidence from prospective studies that childhood UI is associated with increased levels of emotional problems in adolescence (Feehan et al., 1990; Fergusson and Horwood, 1994). Prospective studies have also found evidence that psychological problems in early childhood precede UI at school age (Joinson et al., 2016; Joinson et al., 2019). A recent longitudinal study of a community sample found evidence for bidirectional associations, with psychological problems (including anxiety and depressive symptoms), being both risk factors and outcomes of childhood bedwetting (Kessel et al., 2017). Collectively, the evidence suggests that psychological problems in early childhood increase the risk of UI and that childhood UI leads to increased levels of psychological distress. No prospective cohort studies, however, have examined if changes in emotional problems over time are related to childhood UI, so this remains an unresolved issue.

The current study builds on and extends previous research by advancing understanding of the relationship between emotional problems and UI during childhood using data from a large UK birth cohort. Specifically, the aims of the study are to examine (i) whether the presence of UI in children is related to changes in their emotional problems over time and (ii) whether changes in emotional problems over time are related to the subsequent risk of UI.

## 2 Methods

### 2.1 Participants

Data were obtained from the Avon Longitudinal Study of Parents and Children (ALSPAC) – a large UK-based prospective cohort. Pregnant women resident in Avon, UK with expected dates of delivery between 1st April 1991 and 31st December 1992 were invited to take part in the study. 20,248 pregnancies have been identified as being eligible and the initial number of pregnancies enrolled was 14,541. Of the initial pregnancies, there was a total of 14,676 foetuses, resulting in 14,062 live births and 13,988 children who were alive at 1 year of age (Boyd et al., 2013; Fraser et al., 2013). When the oldest study children were approximately 7 years-old, an attempt was made to increase the sample with participants who failed to enroll during original recruitment. The total sample size for analyses using any data collected after the age of seven is therefore 15,447 pregnancies, resulting in 15,658 foetuses (Northstone et al., 2019). The study website contains details of all data that is available through a fully searchable data dictionary and variable search tool (http://www.bristol.ac.uk/alspac/researchers/our-data/). Ethical approval was obtained from the ALSPAC Ethics and Law Committee and the Local Research Ethics Committee. Informed consent for use of data collected via questionnaires and clinics was obtained from participants following the recommendations of the ALSPAC Ethics and Law Committee at the time.

### 2.2 Childhood urinary incontinence

At 7½ and 9½ years parents were asked “How often does your child wet him/herself during the night/day?” The response options comprised 6 categories: ‘Never’; ‘Occasional accident but less than once a week’; ‘About once a week’; ‘2-5 times a week’; ‘Nearly every day’; and ‘More than once a day’. We dichotomised the variables to indicate any level of daytime wetting or bedwetting irrespective of frequency. We conducted additional analyses where we combined the two measures to classify children into one of four categories: ‘dry’, ‘daytime wetting alone’, ‘bedwetting alone’, ‘combined (day and night) wetting’.

### 2.3 Childhood emotional problems

Parents completed the Strengths and Difficulties Questionnaire (SDQ, Goodman, 2001) when their child was aged 6¾ years and again at age 9½ years. We used the five items of the SDQ emotional problems subscale, which assesses symptoms of anxiety and depression. The internal consistency of the emotional problems subscale of the Strengths and Difficulties Questionnaire has previously been reported; omega values for emotional problems at ages 6¾ years and 9½ years were 0.78 and 0.82 respectively (Speyer et al., 2023).

### 2.4 Statistical modelling

We used a latent difference score (LDS) model (McArdle, 2009), within the analytical framework of structural equation modelling (SEM). Full details of the LDS model, including the main assumptions, are provided in the Supplementary Material. The main advantage of the LDS approach is that by treating the theoretical construct as a latent trait, we can separate ‘true’ change from that due to measurement error present within the manifest items. We can also test for measurement invariance, since changes in the item properties may be another reason why differences in a measure are observed between two waves of data collection. The LDS model permitted us to examine changes in emotional problems between 6¾ and 9½ years and UI status at both 7½ and 9½ years. All models were estimated using Mplus v.7.11 (Muthén and Muthén, 2012) and the WLSMV (weighted least square mean and variance adjusted) estimator in Mplus. WLSMV allows missingness to be only a function of observed covariates and not observed outcomes (Asparouhov and Muthén, 2006). The use of WLSMV means that the restriction to our samples is governed primarily by the explanatory variables in the model. Within any of the samples defined by the availability of explanatory variable(s) it is possible to estimate the model if there is at least some data available on the dependent variable(s).

#### 2.4.1 Magnitude of change in emotional problems

**Fig. 1:**
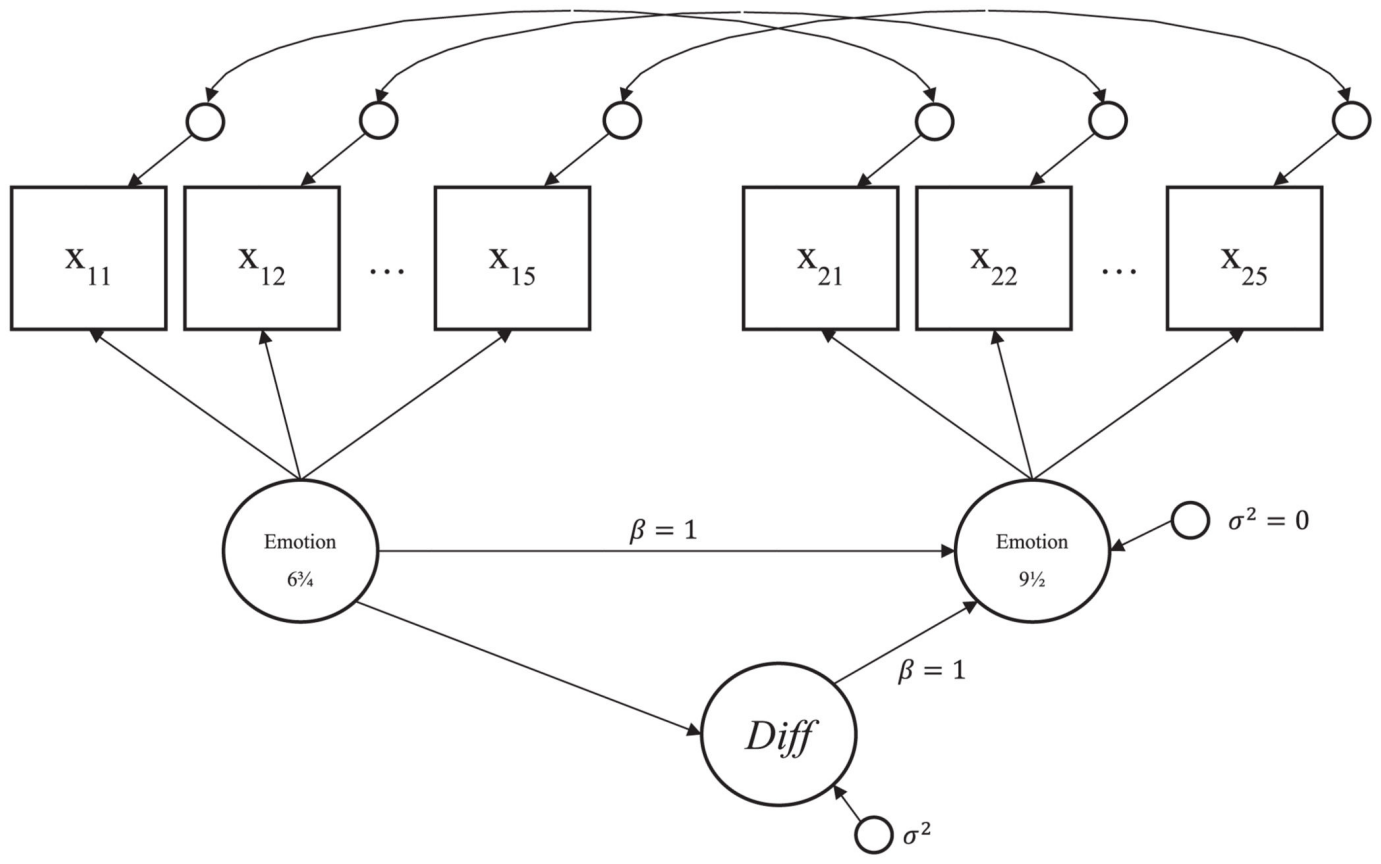
Latent Difference Model used to derive latent variable “*Diff*” representing change in emotional problems between 6¾ and 9½ years.

The basic LDS model is shown in Fig. 1. We used latent trait models with ordinal indicators to derive a pair of latent variables that capture cross-sectional variation in levels of emotional problems at 6¾ and 9½ years. A third latent variable that we refer to as “*Diff*” describes *change* in emotional problems across the time-period (6¾ to 9½ years). Measurement invariance applied to the two trait models ensures that *Diff* captures ‘true’ change (i.e. change adjusted for measurement error) rather than changes in the item properties. This allows us to test hypotheses about (a) the magnitude of change over time, (b) the main covariates of the level of change and (c) consequences of the change. *Diff* was subsequently used as either the dependent or independent variable in the remaining analyses (further details below).

#### 2.4.2 UI and change in emotional problems

We examined whether UI status at age 7½ is associated with a change in emotional problems. Estimates are expressed as (standardized) mean differences in *Diff* across UI categories. Models were adjusted for the child’s sex assigned at birth, maternal educational attainment (high school qualifications or greater vs. certificate of secondary school / vocational/none), stressful life events score at 42 months, and the other SDQ subscales at 6¾ years (sum-scores for prosocial behaviour, conduct problems, hyperactivity and peer problems). We adjusted for these variables because there is robust evidence that they are risk factors for both urinary incontinence (e.g. Joinson et al., 2019) and emotional problems (e.g. Essex et al., 2006).

#### 2.4.3 Consequences of change in emotional problems for subsequent UI

We also examined whether a change in emotional problems (*Diff*) is associated with UI at 9½ years. Since our outcome variable was binary, and we were working with the least-squares estimator, we used probit regression to examine this association and adjusted this model for the confounders (listed above). We examined this model in the full sample and also in the subgroup of children with UI at age 7½ years. In the subgroup model we examine whether a change in emotional problems is associated with a continuation or resolution of UI. Models were adjusted for the confounders described above. In the final model we also adjusted for the severity of UI at age 7½ using a variable indicating whether children experienced no wetting, either bedwetting *or* daytime wetting, or combined (day and night) wetting at a frequency of two or more times per week.

## 3 Results

Data on UI (bedwetting and/or daytime wetting) were available for 8188 children at age 7½. Participants with at least one available UI data point were included in the sample. We excluded participants with missing data on both bedwetting and daytime wetting. Table 1 shows the proportion of girls and boys who experienced one or more type of UI at age 7½ and also provides details on maternal educational level, stressful life events and scores on the prosocial behaviour, conduct problems, hyperactivity and peer problems subscales of the SDQ.

At age 9½ the questions on childhood UI were repeated and 6864 parents responded. Parents of 83.6 % children with bedwetting at age 7½ also provided data on their child’s wetting at age 9½. After excluding cases with missing data on both questions the data shows that at age 9½ years, 87.0 % of children were dry, 8.1 % had bedwetting alone, 3.3 % had daytime wetting, and 1.7 % had combined (day and night) wetting.

### 3.1 Magnitude of change in emotional problems

The goodness of fit of this model was satisfactory and the indicators were highly loaded on common factors. Comparison of means and intercepts for both common factors showed that, on average, the level of emotional problems decreased from 6¾ to 9½ years by 0.042 standard deviations (SDs). The coefficient for *Diff* ON *Emotion 6¾* (− 0.338; *p* < 0.001) in Fig. 1 indicates that children with more emotional problems at age 6¾ experience a decrease in emotional problems over time.

### 3.2 UI and change in emotional problems

We examined whether the presence of UI (bedwetting, daytime wetting or both (1); reference category (0) = no urinary incontinence) at age 7½ is related to the magnitude of change in emotional problems from 6¾ – 9½ years. The results for these models are shown in table S1 in the supplement. Compared to children with no UI (ref cat = 0) at age 7½, those with UI (1) had, on average, 0.264 SD (SE = 0.047, *p* < 0.001) higher scores on *Diff* variable. Higher values on the *Diff* factor resulted in higher scores on the emotional problems factor at 9½ years. This association remained in the fully adjusted model (beta = 0.146, p < 0.00).

We also examined the effect of different types of UI on change in emotional problems by creating three dummy variables to indicate whether children at age 7 had daytime wetting alone, bedwetting alone, or both types of UI (reference category = no UI at age 7½). The results in Table 2 provide evidence that children with bedwetting or daytime wetting alone experienced a change (increase) in emotional problems over time and the magnitude of the effect is similar for each type of urinary incontinence. Among children with *both* bedwetting and day-time wetting, the increase in emotional problems over time was over twice the magnitude of that seen in children with a single type of urinary incontinence. The results remained unchanged after the model was adjusted for confounders (sex, mother’s education, stressful events and other SDQ subscales).

### 3.3 Consequences of change in emotional problems for subsequent UI

We examined whether the change in emotional problems from 6¾ – 9½ years is related to the probability of UI at age 9½ (Table 3). The Probit coefficients show the positive effects of an increase in emotional problems on the probability of UI at 9½ years. Fig. S1 (a) is provided to aid interpretation of the effect of the *Diff* variable on the probability of UI at age 9½. The figure shows that for children without any change in emotional problems (*Diff* =*0*) the probability of UI at age 9½ is low (close to 10 %) and for children with a *decrease* in emotional problems the expected probability of UI at age 9½ is even lower (around 5 %). The results also provide evidence that an increase in emotional problems from 6¾ – 9½ years is associated with an increased probability of UI at age 9½. For a one SD increase in emotional problems (*Diff* of 1 SD) the expected probability of UI at 9½ years is 25 %; for a *Diff* of 2 SD the probability of UI is above 40 % and for *Diff* of 3 SD the probability of UI is nearly 60 %. The reported effect remained after adjusting for confounders.

Finally, we examined the effect of a change in emotional problems on UI in the subgroup of children who had UI at 7½ years (Table 3 and Fig. S1 (b)). There was evidence for an effect in the unadjusted model, but this was attenuated in the fully adjusted model.

## 4 Discussion

Children with UI at age 7½ experienced an increase in emotional problems from 7½ to 9½ years compared to children who were dry. The increase in emotional problems was greater in children with combined (day and night) wetting compared to children with either daytime wetting or bedwetting alone. We also found that a change in emotional problems over time (from age 6¾ to 9½ years) is associated with the probability of experiencing subsequent UI at age 9½.

Major strengths of this study are the use of data from a large birth cohort, and the prospective design which allowed us to examine changes in emotional problems over time in relation to childhood UI. We did not restrict our analysis to children who met clinical diagnostic criteria, therefore, the study findings apply to children in the community and not just to those with UI that meets clinical diagnostic criteria. Parental reports of emotional problems are a potential limitation since parents of children with UI may more readily identify emotional problems in their child, especially if they believe this is a cause of their child’s wetting. In earlier prospective studies using data from the ALSPAC cohort, however, higher levels of parent-reported emotional problems at age 3 were associated with UI at school age (i.e., before parents generally consider UI to be unusual or problematic) (Joinson et al., 2016; Joinson et al., 2019).

Several limitations of our study should be considered when interpreting the findings. We used data on UI and emotional problems at only two time points in childhood and the most proximal measure of emotional problems was at 6¾ years, when ideally it would have been at the same time as UI (at age 7½ years). Further research is needed with repeated measures of UI and emotional problems across multiple time points to examine the interrelationship between these variables over time. There was no information available on underlying organic causes of UI in our sample, but most cases of bedwetting and daytime wetting in children and adolescents are known to be functional (von Gontard and Nevéus, 2006). We did not consider whether treatment for UI might have impacted on the findings. Parents were asked to report whether children had received treatment for bedwetting (alarm or medication) at ages 7½ and 9½ years, but only a small proportion of children (0.2 % – 0.4 %) were treated and there was no information on onset or duration of treatment.

The ALSPAC sample is predominantly White and affluent and therefore, we are unable to generalise our findings to minority ethnic groups and less affluent populations. Further research in these under-served populations is vital to prevent widening inequalities in heath research.

### 4.1 Potential mechanisms explaining the findings

The increases in emotional problems over time in children with UI could be due to children’s growing awareness that UI is unusual for their age, or in the face of negative reactions from their parents or peers. There is evidence that children with UI experience low self-esteem and reduced quality of life (Hägglöf et al., 1998; Bower, 2008; Gladh et al., 2006), which are associated with an increased risk of emotional problems in children (Keane and Loades, 2017; Sharpe et al., 2016). Children with combined (day and night) wetting experienced a greater increase in emotional problems than those with either daytime wetting or bed-wetting alone. This could be because children with combined wetting experienced more frequent (severe) wetting than those with bedwetting or daytime wetting alone (25 % of children with combined wetting at age 7½ experienced bedwetting twice or more per week compared with 15 % of those with bedwetting alone. 17 % of children with combined wetting experienced daytime wetting twice a day or more compared with 9 % of those with daytime wetting alone).

We also found evidence that a greater increase in emotional problems between the ages of 6¾ to 9 ½ years is associated with a greater probability of UI at age 9½. Emotional problems are associated with increased psychological stress, which has been found in animal studies to increase voiding frequency and bladder dysfunction (Gao and Rodríguez, 2022).

## 5 Conclusions

Using a latent difference score model, our study makes a unique contribution to the literature by finding evidence that primary school-age children with UI experience increases in emotional problems over time. An important clinical implication of our findings is that emotional problems should be evaluated and monitored in children being treated for UI and that particular attention should be paid to children with both daytime wetting and bedwetting since they experience the greatest increase in emotional problems over time. With increasing age, UI becomes more socially unacceptable, and parents show more intolerance (Butler and McKenna, 2002). It is important to address these possible causes of emotional problems in children with UI by providing support to children experiencing problems managing their incontinence at school (e.g. lack of access to school toilets, social exclusion and peer victimisation) (Whale et al., 2018) and by emphasising to parents that children are not to blame for their incontinence. We found that changes in emotional problems over time were associated with the probability of experiencing subsequent UI. Intervening to prevent or reduce emotional problems could, therefore, decrease the probability that UI will continue into later childhood and adolescence. The presence of UI in mid-adolescence is associated with an increase in mental disorders in later adolescence (Gordon et al., 2023). Early intervention is crucial to prevent childhood UI from becoming persistent and impacting on young people’s mental health.

Further research is needed to determine whether relationships between emotional problems and UI are due to causal effects (and whether these effects are bidirectional), and to identify mechanisms. Non-causal relationships between emotional problems and UI are still important because the presence of mental health issues can affect treatment outcomes (Van Herzeele et al., 2015).

Parents should be encouraged to seek help if their child is still wetting frequently in the day and/or night at age 5 because there is evidence that successful treatment for childhood UI leads to increased health-related quality of life and improvements in self-esteem (Hägglöf et al., 1998; Longstaffe et al., 2000; Equit et al., 2014).

## Supplementary Material

Appendix A. Supplementary data Supplementary data to this article can be found online at https://doi.org/10.1016/j.jad.2025.04.020.

## Figures and Tables

**Table 1:** Characteristics of participants in the total sample (*n* = 8188) and among each type of UI problem at 7½ years.

	Dry	Daytime wetting alone	Bedwetting alone	Bedwetting and daytime wetting	Total
*Sex*					
Girls: n (%)	3326 (50.7 %)	233 (62.5 %)	300 (30.3 %)	118 (43.9 %)	3977 (48.6 %)
Boys: n (%)	3231 (49.3 %)	140 (37.5 %)	689 (69.7 %)	151 (56.1 %)	4211 (51.4 %)
*Maternal education*					
Certificate of secondary school/vocational/none: n (%)	1497 (23.6 %)	77 (21.2 %)	217 (22.5 %)	59 (22.6 %)	1850 (23.3 %)
High school qualifications and above: n (%)	4861 (76.5 %)	287 (78.9 %)	748 (77.5 % 0	202 (77.4 %)	6098 (76.7 %)
*Stressful life events score at 42 months*					
Mean (SE)	1.371 (0.018)	1.553 (0.081)	1.506 (0.052)	1.820 (0.122)	1410 (0.017)
*Strengths and difficulties subscale scores at 7½ years – mean (SE)*					
Prosocial behaviour	8.223 (0.022)	8.124 (0.094)	7.856 (0.063)	7.919 (0.132)	8.165 (0.021)
Conduct problems	1.514 (0.019)	1.867 (0.079)	1.813 (0.051)	2.034 (0.097)	1.583 (0.017)
Hyperactivity	3.208 (0.030)	3.852 (0.129)	3.790 (0.084)	4.245 (0.163)	3.341 (0.028)
Peer problems	0.992 (0.018)	1.355 (0.092)	1.219 (0.053)	1.212 (0.105)	1.043 (0.017)

**Table 2:** Association between different types of UI at age 7½ and change in emotional problems from 6¾– 9½ years.

		Unadjusted		Adjusted 1		Adjusted 2		Adjusted 3		Adjusted 4
		β (SE)		β (SE)		β (SE)		β (SE)		β (SE)
Dry		0.00 ref		0.00 ref		0.00 ref		0.00 ref		0.00 ref
Daytime wetting alone		0.205 (0.085)		0.182 (0.085)		0.191 (0.085)		0.208 (0.086)		0.016 (0.027)
Bedwetting alone		0.215 (0.059)		0.262 (0.059)		0.248 (0.059)		0.240 (0.061)		0.050 (0.019)
Both day time and bedwetting		0.535 (0.103)		0.553 (0.102)		0.531 (0.102)		0.508 (0.104)		0.117 (0.033)
		P < 0.001		P < 0.001		P < 0.001		P < 0.001		P < 0.001
Sample size		*N =* 7789		N = 7789		*N =* 7595		*N =* 7196		*N =* 7128

Adjusted 1: adjusted for child’s sex.

Adjusted 2: further adjusted for maternal educational attainment.

Adjusted 3: further adjusted for stressful life events at 42 months.

Adjusted 4: further adjusted for other SDQ subscales.

Effect estimates are standardized mean differences in *Diff* relative to the group of children who are dry at age 7½.

**Table 3:** Association between change in emotional problems from 6¾ to 9½ years and probability of UI at age 9½.*

		Effect of *Diff* on binary measure of UI at 9½ years (estimates are coefficients from probit model)						
		In whole population				In children who have UI at 7½ years		
		N	Est. (SE)	*p*-value		N	Est. (SE)	p-value
Unadjusted		7812	0.145 (0.030)	<0.001		1558	0.112 (0.056)	0.046
Adjusted 1		7812	0.159 (0.030)	<0.001		1558	0.117 (0.056)	0.038
Adjusted 2		7618	0.147 (0.030)	<0.001		1527	0.110 (0.056)	0.051
Adjusted 3		7210	0.138 (0.030)	<0.001		1442	0.086 (0.057)	0.127
Adjusted 4		7137	0.113 (0.031)	<0.001		1425	0.071 (0.057)	0.211
Adjusted 5		7210	0.121 (0.031)	<0.001		1442	0.073 (0.058)	0.206

Adjusted 1: adjusted for child’s sex.

Adjusted 2: further adjusted for maternal educational attainment.

Adjusted 3: further adjusted for stressful life events at 42 months.

Adjusted 4: further adjusted for other SDQ subscales.

Adjusted 5: adjusted model 3 also adjusted for severity of incontinence at baseline (i.e. either bedwetting *or* daytime wetting, or combined (day and night) wetting at a frequency of two or more times per week).

* Probit coefficients indicate the effects of an increase in emotional problems on the probability of UI at 9½ years.

## References

[R1] Asparouhov T, Muthén B (2006). Robust chi square difference testing with mean and variance adjusted test statistics. Mplus web notes: No 10.

[R2] Bower WF (2008). Self-reported effect of childhood incontinence on quality of life. J Wound Ostomy Continence Nurs.

[R3] Boyd A, Golding J, Macleod J, Lawlor DA, Fraser A, Henderson J (2013). Cohort profile: the ‘children of the 90s’—the index offspring of the Avon longitudinal study of parents and children. Int J Epidemiol.

[R4] Butler RJ, McKenna S (2002). Overcoming parental intolerance in childhood nocturnal enuresis: a survey of professional opinion. BJU Int.

[R5] Equit M, Hill J, Hübner A, von Gontard A (2014). Health-related quality of life and treatment effects on children with functional incontinence, and their parents. J Pediatr Urol.

[R6] Essex MJ, Kraemer HC, Armstrong JM, Boyce WT, Goldsmith HH, Klein MH, Woodward H, Kupfer DJ (2006). Exploring risk factors for the emergence of children’s mental health problems. Arch Gen Psychiatry.

[R7] Feehan M, McGee R, Stanton W, Silva PA (1990). A 6-year follow-up of childhood enuresis: prevalence in adolescence and consequences for mental health. J Paediatr Child Health.

[R8] Fergusson DM, Horwood LJ (1994). Nocturnal enuresis and behavioral problems in adolescence: a 15-year longitudinal study. Pediatrics.

[R9] Fraser A, Macdonald-Wallis C, Tilling K, Boyd A, Golding J, Davey Smith G (2013). Cohort profile: the Avon longitudinal study of parents and children: ALSPAC mothers cohort. Int J Epidemiol.

[R10] Gao Y, Rodríguez LV (2022). The effect of chronic psychological stress on lower urinary tract function: an animal model perspective. Front Physiol.

[R11] Gladh G, Eldh M, Mattsson S (2006). Quality of life in neurologically healthy children with urinary incontinence. Acta Paediatr.

[R12] von Gontard A, Nevéus T (2006). Management of Disorders of Bladder and Bowel Control in Childhood.

[R13] von Gontard A, Baeyens D, Van Hoecke E (2011). Psychological and psychiatric issues in urinary and fecal incontinence. J Urol.

[R14] Goodman R (2001). Psychometric properties of the strengths and difficulties questionnaire. J Am Acad Child Adolesc Psychiatry.

[R15] Gordon K, Warne N, Heron J, von Gontard A, Joinson C (2023). Continence problems and mental health in adolescents from a UK Cohort. Eur Urol.

[R16] Hägglöf B, Andrén O, Bergström E, Marklund L, Wendelius M (1998). Self-esteem in children with nocturnal enuresis and urinary incontinence: improvement of self-esteem after treatment. Eur Urol.

[R17] Joinson C, Sullivan S, von Gontard A, Heron J (2016). Early childhood psychological factors and risk for bedwetting at school age in a UK cohort. Eur Child Adolesc Psychiatry.

[R18] Joinson C, Grzeda MT, von Gontard A, Heron J (2019). A prospective cohort study of biopsychosocial factors associated with childhood urinary incontinence. Eur Child Adolesc Psychiatry.

[R19] Keane L, Loades M (2017). Review: low self-esteem and internalizing disorders in young people - a systematic review. Child Adolesc Ment Health.

[R20] Kessel EM, Allmann AE, Goldstein BL, Finsaas M, Dougherty LR, Bufferd SJ, Carlson GA, Klein DN (2017). Predictors and outcomes of childhood primary enuresis. J Am Acad Child Adolesc Psychiatry.

[R21] Longstaffe S, Moffatt ME, Whalen JC (2000). Behavioral and self-concept changes after six months of enuresis treatment: a randomized, controlled trial. Pediatrics.

[R22] McArdle (2009). Latent variable modeling of differences and changes with longitudinal data. Annu Rev Psychol.

[R23] Muthén LK, Muthén BO (2012). Mplus User’s Guide.

[R24] Northstone K, Lewcock M, Groom A (2019). The Avon Longitudinal Study of Parents and Children (ALSPAC): an update on the enrolled sample of index children in 2019. Wellcome Open Res.

[R25] Sharpe H, Patalay P, Fink E, Vostanis P, Deighton J, Wolpert M (2016). Exploring the relationship between quality of life and mental health problems in children: implications for measurement and practice. Eur Child Adolesc Psychiatry.

[R26] Speyer LG, Auyeung B, Murray AL (2023). Longitudinal invariance of the strengths and difficulties questionnaire across ages 4 to 16 in the ALSPAC sample. Assessment.

[R27] Thibodeau BA, Metcalfe P, Koop P, Moore K (2013). Urinary incontinence and quality of life in children. J Pediatr Urol.

[R28] Van Herzeele C, De Bruyne P, De Bruyne E, Walle JV (2015). Challenging factors for enuresis treatment: psychological problems and non-adherence. J Pediatr Urol.

[R29] Whale K, Cramer H, Joinson C (2018). Left behind and left out: the impact of the school environment on young people with continence problems. Br J Health Psychol.

