# Supplementary material for: Emotional problems and urinary incontinence in children from a UK cohort
Source: J Affect Disord. Author manuscript; Available in PMC 2026 Apr 7. (PMC7618977; doi:10.1016/j.jad.2025.04.020)
Supplement: Appendix A. Supplementary data — Supplementary data to this article can be found online at https://doi.org/10.1016/j.jad.2025.04.020. [file EMS212363-supplement-Appendix_A__Supplementary_data.docx]

**Supplementary materials**

**Main assumptions underlying the LDS model**

*1) Decomposition of the variable score over time*. The fundamental assumption of the LDS model states that the score on modelled variable *y* at the time-point *t* can be decomposed into two parts. First part is the score on variable *y* at previous time point (*t-1*) and the second is the difference in score (*Δy_t_*) between *t-1* and *t*. Thus, the score on variable y at time point *t* is defined as: $y_{t}=y_{t-1}+{\Delta y}_{t}$. Thus, the score at time *t* is operationalized as the one consisting of the part passed from (or inherited from) the baseline time point and the second part accounted for by the pure change in scores between both moments.

*2) Modelling latent difference score within SEM.*  By putting the LDS model into an SEM context, it is possible to model the difference score both as an outcome and exposure and to evaluate the overall fit of any specific theoretical model against the data and taking into account measurement error.

3) A key advantage of the LDS models is that it enables modelling the pattern of change of both observable variables and latent variables (conceptualized as common (latent) factors with several manifest indicators) [1]. A schematic illustration of both approaches to latent difference is described in the figures below. Figure (a) for modelling change in observable variables and figure (b) for modelling change in a latent variable framework.

| (a) Latent difference model for observed variables | (b) Latent difference model for latent variables |
| --- | --- |
| 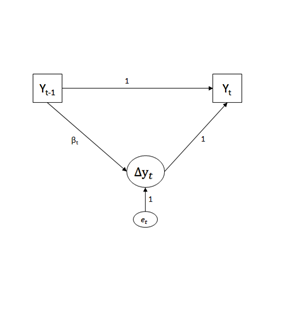 | 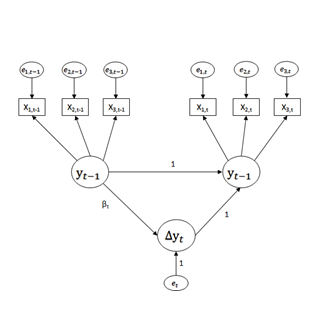 |

## ***Plan of the analyses: development of the model for determinants and consequences of UI in 4 stages***

In the current study we modelled the dynamic of emotional problems among children between age 6 years and 9 months and 9 years and 7 months to determine its relation to UI at 7 years and 7 months and 9 years and 7 months (see Measures section for details). The modelling process was split into 4 stages, each focusing on different aspects and time points of investigated relation. At each stage our models we started from the simplest version including only key variables under interest and then including confounders in each successive step.

***Stage1***

At Stage1 we established the baseline LDS model. This included only the measurement part and established the factorial measurement model of emotional problems and explored the issue of longitudinal invariance. In that model the measure of emotional problems was deemed as a latent (common) factor operationalized by 5 manifest indicators (see Measures section for detailed list of indicators). Once the validity of this measurement model at both time points separately was confirmed, we then examined its stability over time. To do so, a single longitudinal factor model was applied including indicators from both time points. In this model, the factor loadings and the item thresholds^[[1]](#footnote-1)^ of the same indicators at 6y9m and 9y7m were constrained to be the same. Also, because it was longitudinal model the residual correlations of pairs of the same indicators in different time points were allowed to be correlated. Additionally, we decided to test the measurement model constraining these values to be the same for all pairs of indicators. Finally, when the longitudinal equivalence of the measurement model has been established (thus proving that the results on the factor measuring emotional problems are directly comparable at both time points), we expanded the model to include the latent difference score variable, as yet an additional unobserved variable named *diff*. It was defined as latent score and conceptualized as the ‘pure’ amount of change in psychological problems between both time points (6y9m and 9y7m). The aim of this model was to explore general pattern of changes in emotional problems over time and check whether it fits the data reasonably well.

***Stage2***

When the initial model was successfully established it was further expanded into the model in which the latent *diff*erence factor (here change in emotional problems) was modelled along with its determinants/covariates and distant consequences. At the first instance determinants/covariates of latent *diff*erence were tested. We examined the impact of incontinence on changes in emotional problems in two ways. First, we included in our baseline model the information describing incontinence at 7y7m in a form of dichotomous variable indicating presence of any UI (1) versus none (0). In the second approach, we tested the impact of changes in UI on psychological problems by means of 3 dummy variables, each describing a specific type of UI (daytime wetting alone, bedwetting alone, both problems) and dryness at 7y7m as a reference category.

Once both models have been established and validated, in the next step the model was adjusted for the list of potential confounders (see the section: *Confounders* for detailed list of variables included in the model). The confounders were added to the model as other predictors/risk factors of the variable describing latent *diff*erence in the following order: sex, mother’s education, life events and other psychological and behavioural *diff*iculties derived from ALSPAC SDQ datasets (*prosocial behaviour score*, *conduct problems score*, *hyperactivity* and *peer problems* *score)*.

***Stage3***

In the next stage the focus of the analyses was to model the consequences of change in emotional problems (between 6y9m and 9y7m) on subsequent UI at (9y7m). Since the outcome variable was binary (with value 1 representing presence of UI of any kind and 0 representing no UI) the relation between change on emotional problems variable and UI at 9y7m was modelled as *probit* regression.

The *probit* model is a member of wider family of general linear models (GLM) [2] with the link function that ties expected values of the (dichotomous) outcome variable with the standardized normal distribution (z-scores) being modelled as a function of predictors and error term. The modelling process in *probit* regression returns linear coefficients that show how much change in z-score is expected with one unit change of the predictor. The results are frequently translated into marginal probability effects showing how change in each unit of explanatory variable affects predicted probability of the outcome. For convenience this relation can be presented graphically as shown figure S1. Another standard way of dealing with *probit* regression coefficients is to approximate logistic coefficients from them by using standard multiplicator of 1.8. Proxies of logistic regression coefficients can easily be transformed into odds ratios (ORs) [2]. We also used that form in our presentation. The model developed at this stage was adjusted for the same list of confounders as used at Stage3.

***Stage 4***

Finally, the model developed at the previous stage was repeated for a subgroup of children with a UI at age of 7y7m. The group was distinguished on the basis of a dichotomous variable specifying whether at the given age the child had UI of any type (bedwetting, daytime wetting or both). If there was any UI present, then the child was included in the subgroup. The reason why an LDS model was implemented for the subgroup was to check whether there are any peculiarities for that group of children in the pattern of relations between changes on emotional problems and further UI (at age 9½ y7y). Similarly, to previous models we also included the confounding variables.

***The issue of model fit***

All structural models tested in this paper were estimated within Mplus v.7.11 [3]. Since all the observable indicators of the measurement model of emotional problems were categorical, the WLSMV estimation method was chosen [4]. The evaluation of the models with regards to goodness of fit was based on two statistics [4]. As a first index of fit we included Root-Mean-Square-Error-of-Approximation (RMSEA) described out by Steiger and Lind (1980) [5]. This index includes adjustment to parsimony and reports the overall model fit. RMSEA values of 0.08 or less usually indicate acceptable fit. Values above 0.10 are deemed as unacceptable. Highly desirable values lie near 0.06 or below [6]. The cut-off value of 0.06 is however known to work very well when all variables included in the model are continuous, normally distributed and the sample size is large. For other settings, such as when the data are categorical and /or the sample is small, the application of the threshold should be less strict. The reason for this is that there has been a tendency to over-reject well specified models [7]. According to simulation studies, when the indicators are categorical and sample size is small a cut-off point set at 0.09 gives a reasonable rate of rejections of models [7].

Another index of goodness of fit of the model that we took into account was comparative fit index (CFI) [8]. This index compares a proposed model with the baseline model, representing a null model which assumes no relations among the items (all co-variances /correlations assumed to be zero). According to the literature values of CFI indicating a good fit of the model to the data are 0.90 or above. More recently however Hu and Bentler [6] suggested a cutoff point at the level of 0.95. CFI at this level or above indicates a good model fit. We decided here to adapt less strict criterion of 0.90.

Another criterion that we considered in our evaluation of the confirmatory factor model is called convergent validity of items to the construct. This criterion refers to the significance (at alpha level =0.05) of parameter estimates and the magnitude of estimates. According to this rule the items that do not have significant loadings are candidates for deletion. A rule of thumb related with this criterion adapted here states that the standardized loadings should be in the range of 0.30 to 0.90. (Loadings above 0.90 are not optimal as they can be indicative of item redundancy and frequently result in correlated measurement error which lowers the overall model fit and from a conceptual point of view also ‘threatens’ the unidimensionality of given factor).

Supplementary references

1. McArdle 2009 Latent Variable Modeling of *Diff*erences and Changes with Longitudinal Data. Annu. Rev. Psychol, 60:577–605
2. Agresti, A. (2002). *Categorical Data Analysis* (Second Edi). John Wiley & Sons, Inc
3. Muthén LK, Muthén BO. Mplus User's Guide. 7th Edition: Los Angeles, California: Muthén & Muthén, 2012.
4. Brown, T. A. (2006). *Confirmatory Factor Analysis*. The Guilford Press.
5. Steiger, J. H., & Lind, J. (1980). Statistically-based tests for the number of common factors. *Conference Paper*.
6. Hu, L.T., & Bentler, P. (1995). Evaluating Model Fit. In R. H. Hoyle (Ed.), *Structural Equation Modeling: Concepts, issues, and applications* (pp. 76–99). Sage Publications.
7. Yu, C.-Y. (2002). Evaluating Cutoff Criteria of Model Fit Indices for Latent Variable Models with Binary and Continuous Outcomes. *Doctoral Dissertation Submitted and Approved at University of California*.
8. Bentler, P. M. (1990). Comparative fit indexes in structural models. *Psychological Bulletin*, *107*(2), 238–246.

Table S1. Association between any UI at age 7 and change in emotional problems from 7 – 9½ years.

|  | N | Beta (SE) | p-value |
| --- | --- | --- | --- |
| Unadjusted | 7,799 | 0.264 (0.047) | <0.001 |
| Adjusted for sex | 7,799 | 0.290 (0.047) | <0.001 |
| Further adjusted for mum-ed | 7,605 | 0.280 (0.047) | <0.001 |
| Further adjusted for life events | 7,206 | 0.274 (0.048) | <0.001 |
| Further adjusted for other SDQ subscales | 7,137 | 0.146 (0.043) | <0.001 |

Figure S1. Predicted probabilities (along with the 95%CI) of UI at 9 as a function of changes on psychological problems variable *Diff* (standardized). Projection obtained from unadjusted model.

1. Whole population


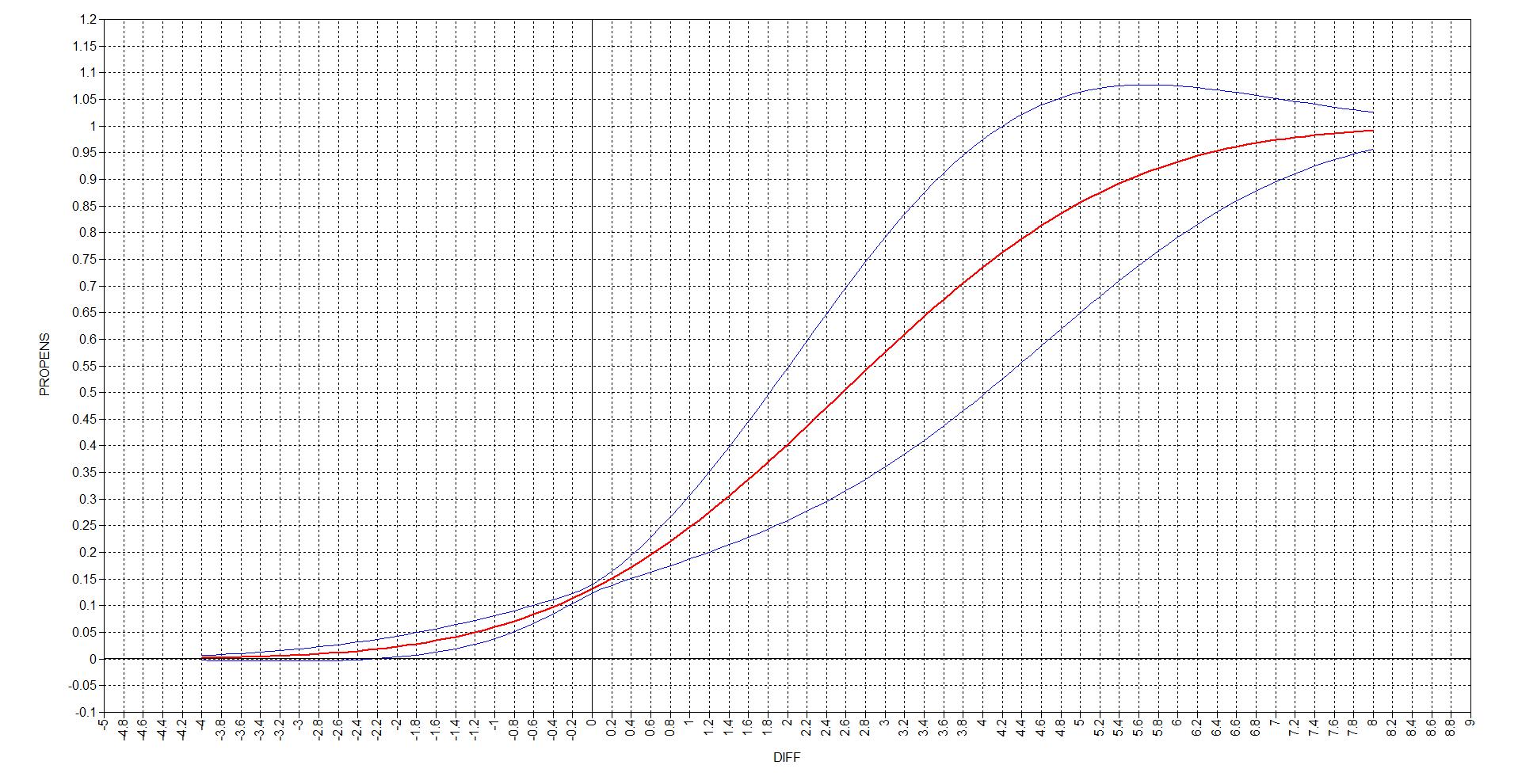


1. Subgroup who have UI at 7½ years


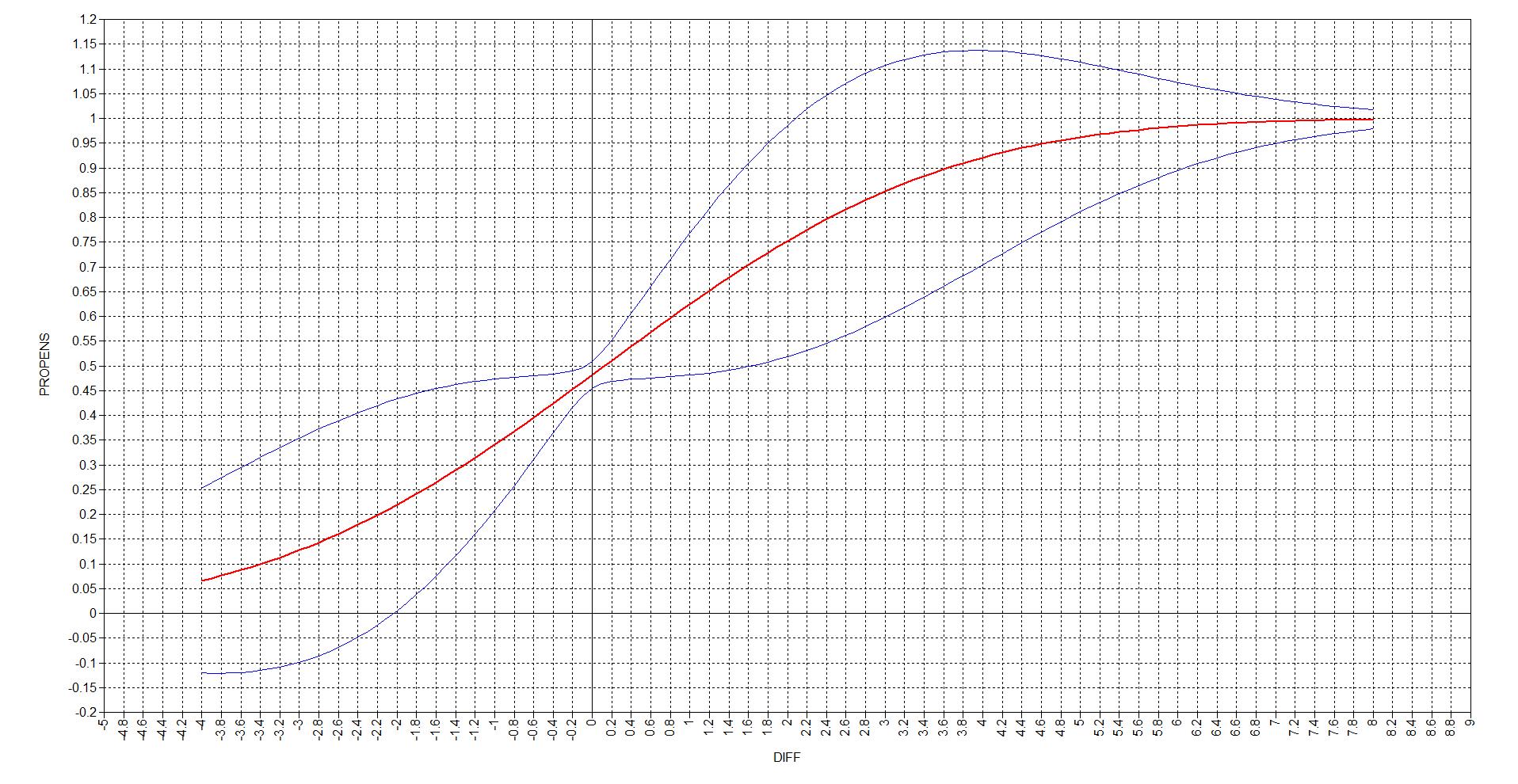


1. Thresholds were included in the models since the indicators were categorical variables [↑](#footnote-ref-1)
